# Association of Race and Ethnicity with Genomic Testing at a Comprehensive Cancer Center in North Carolina

**DOI:** 10.1158/2767-9764.CRC-24-0134

**Published:** 2024-11-18

**Authors:** Clare Meernik, Frances Wang, Yadurshini Raveendran, Michelle F. Green, Devon K. Check, Hayden B. Bosworth, Linda M. Sutton, John H. Strickler, Tomi F. Akinyemiju

**Affiliations:** 1 Department of Population Health Sciences, Duke University School of Medicine, Durham, North Carolina.; 2 Duke Cancer Institute, Duke University School of Medicine, Durham, North Carolina.; 3 Department of Pathology, Duke University School of Medicine, Durham, North Carolina.; 4 Center of Innovation to Accelerate Discovery and Practice Transformation, Durham Veterans Affairs Medical Center, Durham, North Carolina.; 5 Division of General Internal Medicine, Department of Medicine, Duke University Medical Center, Durham, North Carolina.; 6 Department of Psychiatry and Behavioral Sciences, Duke University Medical Center, Durham, North Carolina.; 7 Duke University School of Nursing, Duke University School of Medicine, Durham, North Carolina.; 8 Duke Cancer Network, Duke Cancer Institute, Durham, North Carolina.

## Abstract

Genomic testing, which analyzes tumor cells or circulating tumor DNA to guide cancer treatment, is increasingly used in precision oncology. However, data on racial and ethnic disparities in its use are limited. This retrospective cohort study included patients diagnosed with stage IV breast cancer, colorectal cancer, non–small cell lung cancer, or prostate cancer in 2014 to 2019. Patients were identified from an institutional multilevel data warehouse, and their data were linked to an institutional genomics database and electronic health records. Multivariable logistic regression was used to estimate OR and 95% confidence interval (CI) for the receipt of tumor-specific genomic testing among non-Hispanic (NH) Black and NH White patients, stratified by cancer type. The linked dataset included 3,461 patients, 44.5% of whom received genomic testing. NH Black patients with prostate cancer were less likely to use genomic testing versus NH White patients across the study period (fully adjusted OR, 0.55, 95% CI, 0.32–0.95), as were NH Black patients diagnosed with breast cancer in 2014 to 2016 (OR adjusted for patient-level covariates: 0.34, 95% CI, 0.11–0.99). No differences in testing were observed between NH Black and NH White patients with colorectal or non–small cell lung cancer or breast cancer diagnosed in 2017 to 2019. Lower documented use of tumor-specific genomic testing among NH Black patients with prostate cancer across the study period underscores the need to monitor trends and address disparities in the use of emerging precision oncology technologies. Future work targeting multilevel barriers to genomic testing is a crucial next step forward.

**Significance:**

Non-Hispanic Black patients diagnosed with prostate cancer between 2014 and 2019 and treated at a comprehensive cancer center were less likely to use tumor-specific genomic testing compared with non-Hispanic White patients. Disparities in the use of precision oncology technologies should be monitored and addressed to ensure equitable cancer care.

## Introduction

Persistent and, in some cases, worsening racial disparities in cancer mortality constitute a public health crisis in the US. Common cancers (e.g., breast, colorectal, lung, prostate) all demonstrate significant racial disparities in outcomes: Black patients experience up to two times the risk of dying from their cancer (1), and 5-year survival remains worse even after accounting for stage at diagnosis (2).

There is growing concern that these disparities in cancer mortality will persist and ultimately widen, given emerging evidence of inequities in genomic testing, which may lead to disparate trial participation and lesser use of biomarker-directed (i.e., targeted) therapies for Black patients (3). Tumor-specific genomic testing analyzes tumor cells or circulating tumor DNA isolated from blood to identify mutations or other genomic changes that drive specific cancers, leading to the selection of targeted therapies that precisely match the tumor’s specific mutations (4). Genomic testing and targeted therapies have transformed the landscape of cancer treatment in recent years, leading to less side effects and improved survival (5). Accordingly, national guidelines [e.g., from the National Comprehensive Cancer Network (NCCN; ref. 6)] now incorporate genomic testing as standard care for some tumor types, and the National Institutes of Health All of Us initiative has highlighted the accelerated development and adoption of targeted cancer therapies as a national priority (7).

Technical advancements in genomic testing have led to growing use specifically of next-generation sequencing (NGS) approaches that that can test multiple genes simultaneously and are more sensitive for the detection of relevant biomarkers than single-gene PCR-based testing (4). NGS panels vary in size and include smaller “hotspot” panels that only target certain gene regions known to be recurrently mutated in cancer (hotspot NGS), as well as more comprehensive panels that cover all exons of several hundred genes and report genomic signatures such as microsatellite instability and tumor mutational burden [comprehensive genomic profiling (CGP)]. The use of NGS can improve access to targeted therapies (4), increase enrollment in clinical trials (3), and, for some tumor types, lengthen cancer survival (8). During our study years, hotspot NGS and CGP were emerging as one of multiple options for genomic testing, in addition to single-gene testing.

Recent research has shown that Black patients with cancer are less likely to receive certain tumor-specific genomic tests (9–13), although data are limited on disparities for NGS approaches (3, 14). If not addressed, disparities in genomic testing may contribute to increasing disparities in clinical trial participation due to trial eligibility growing increasingly dependent on biomarker identification (3, 15), prevent Black patients from accessing evidence-based targeted therapies, and limit the creation and production of personalized testing and therapies, ultimately worsening disparities in cancer outcomes. Thus, the aim of our study is to evaluate the differences in the use of tumor-specific genomic testing by race and ethnicity among patients with metastatic breast cancer, colorectal cancer, non–small cell lung cancer (NSCLC), or prostate cancer diagnosed during 2014 to 2019 at a large comprehensive cancer center in North Carolina. By examining a period of data from early in the era of genomic testing and including data on NGS, this study aims to identify emerging disparities in precision oncology that need to be addressed as developments in cancer medicine continue to accelerate.

## Materials and Methods

This was a retrospective cohort study of patients with a first primary metastatic (stage IV) breast cancer, colorectal cancer, NSCLC, or prostate cancer; these cancers were selected because they had targeted therapies either approved or in clinical development during the study years (16) and exhibit racial and ethnic disparities in outcomes (1). Relevant tumor-specific genomic tests during the study years examined were single-gene testing (i.e., *KRAS*, *NRAS*, and *BRAF* for colorectal cancer; *BRAF*, *KRAS*, *EGFR*, *ALK*, *RET*, and *ROS1* for NSCLC), in-house hotspot NGS testing (for colorectal cancer and NSCLC), and commercial CGP (for all included cancer types). All included tests were either clinically recommended during the study years or were an emerging option used at our institution.

**Figure 1:**
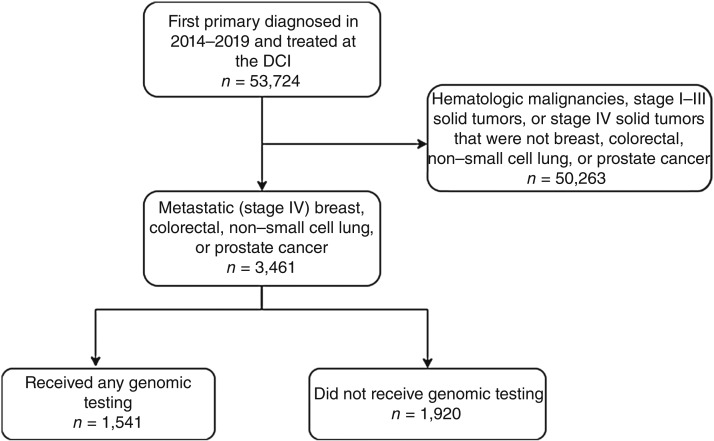
Flow chart of study inclusion. Included participants were patients diagnosed with a first primary metastatic breast cancer, colorectal cancer, NSCLC, or prostate cancer in 2014 to 2019 and treated at the DCI.

Patients were diagnosed between January 2014 and December 2019 at the Duke Cancer Institute (DCI) and were identified from an institutional multilevel data warehouse (DCI CREST) that captures all patients treated at the DCI and integrates data from sources including the Duke Tumor Registry (e.g., sociodemographic characteristics, geographic region, clinical data), Duke Electronic Health Records (EHR), and the National Cancer Institute Healthcare Delivery Research Program Social Determinants of Health (SDOH) by the US Census Tract Dataset [e.g., census tract-level distributions of race and ethnicity, educational attainment, Yost socioeconomic status (SES) index], geocoded based on longitude and latitude of patient residence at diagnosis (National Cancer Institute; RRID:SCR_011176). DCI CREST was linked to the Duke Molecular Registry of Tumors (MRT) using unique Duke medical record numbers. MRT is a secure clinical and research data repository that stores commercial CGP results from patients with cancer within the Duke University Health System who received testing from Foundation Medicine or Guardant Health (17). Panels from Foundation Medicine included FoundationOne, FoundationOne CDX, and FoundationOne Heme for tumor tissue testing and FoundationOne Liquid for ctDNA testing. All testing from Guardant Health involved ctDNA-based Guardant360 panels. The linkage of CREST and MRT provided a dataset with sociodemographic, clinical, and genomic testing data for all patients treated at the DCI with a metastatic breast cancer, colorectal cancer, NSCLC, or prostate cancer (diagnosed 2014–2019) and captured commercial CGP up to mid-2022 (Fig. 1). MRT only captured the use of commercial CGP at our institution, so we additionally linked DCI CREST to the Duke EHR to capture the use of tumor-specific single-gene testing and in-house hotspot NGS panels (relevant only for colorectal cancer and NSCLC during the study years at our institution) and identify the use of genomic testing more broadly. This study was approved by the Duke Health Review Board (Pro00108416). As the research involved secondary data analysis, informed consent was not required. This study was conducted in accordance with the ethical principles of the Belmont Report and the US Common Rule.

Our outcome of interest was the receipt of any tumor-specific genomic testing (commercial CGP, single-gene testing, or in-house hotspot NGS) among patients with metastatic breast cancer, colorectal cancer, NSCLC, or prostate cancer and who were treated at the DCI. Receipt of germline genetic testing was not assessed. Our primary predictor of interest was patient race and ethnicity, which was obtained from the Duke Tumor Registry (self-identified). This analysis focuses specifically on the disparities in genomic testing by race and ethnicity because of the significant racial and ethnic disparities in cancer mortality, particularly among Black patients (18), and the concern that inequities in the use of genomic testing and associated targeted therapies may contribute to persisting or widening survival disparities. In this analysis, race and ethnicity serve as proxy measures for the extent to which social, environmental, and structural factors, including racism, may act as barriers to the use of genomic testing among racially minoritized groups (19). Sociodemographic and clinical covariates were obtained from data sources available within DCI CREST, including the Duke Tumor Registry and the Duke EHR.

### Statistical analysis

Descriptive statistics were used to examine the mean and SD and the median and interquartile range (IQR) of continuous variables and the frequency and proportion of categorical variables. Logistic regression was used to estimate OR and 95% confidence interval (CI) for the receipt of genomic testing by patient race and ethnicity. Unadjusted models were used to estimate the total association of race and ethnicity. Two adjusted models were used to estimate the association of race and ethnicity after controlling for potentially mediating pathways: (i) adjustment for the following patient-level covariates: age at diagnosis (continuous), year of cancer diagnosis (continuous), and insurance status (uninsured, private, Medicaid, Medicare, other insurance), and (ii) adjustment for patient-level covariates as described and the following census tract-level SDOH covariates: rural–urban categorization based on rural–urban commuting area (RUCA) codes (isolated small rural or small rural, large rural/city/town, urban; ref. 20), educational attainment (quintiles of the proportion of the census tract age ≥25 years with a high school education or less), and the Yost SES index—an area-based composite measure of SES (quintiles; ref. 21). Analyses were stratified by cancer type. We report descriptive statistics for patients of any race or ethnicity, although regression analyses only included non-Hispanic (NH) Black and NH White patients due to sample sizes. NH Black and NH White patients missing any covariate data were excluded from regression analysis (*n* = 295; 9.2%).

### Sensitivity analysis

Due to variability in patient eligibility for genomic testing with changing clinical practice guidelines throughout the study period—and to account for the influence of particular patient characteristics on the likelihood of testing referral (e.g., patient prognosis)—we conducted several sensitivity analyses: (i) analysis was stratified by year of diagnosis corresponding to more frequent clinical use of testing in more recent years (2014–2016 vs. 2017–2019), (ii) analysis excluded patients who died within 120 days of diagnosis, and (iii) analysis excluded patients with no record of cancer treatment at our institution.

### Data availability

The data underlying this article cannot be shared due to the privacy of patients included in the study. More information about accessing summary level data from the CREST Dashboard can be found here: https://sites.duke.edu/dcicoee/crest/.

## Results

### Sample characteristics

The linked dataset included 3,461 patients with stage IV breast cancer (11.5%), colorectal cancer (14.5%), NSCLC (53.0%), or prostate cancer (21.0%), including 1,541 (44.5%) patients who received any tumor-specific genomic testing up to mid-2022 (Table 1). The cohort included patients diagnosed with cancer at a median age of 65 (IQR 57–73) years. The cohort was 68.8% NH White, 26.1% NH Black, 2.1% NH Asian, 1.5% NH other races (including American Indian, Alaska Native, Native Hawaiian, Pacific Islander, or another race), and 1.5% Hispanic. Most patients either had Medicare (47.0%) or private health insurance (29.6%).

**Table 1:** Sociodemographic and clinical characteristics of patients with stage IV cancer diagnosed during 2014 to 2019 (*n* = 3,461), overall and stratified by use of genomic testing

	Overall	Any genomic testing	
		No	Yes
	*N* (%)^a^	*N* (%)^a^	*N* (%)^a^
*N*	3,461	1,920 (55.5%)	1,541 (44.5%)
Race and ethnicity			
Hispanic	49 (1.5%)	28 (1.5%)	21 (1.4%)
NH Asian	71 (2.1%)	24 (1.3%)	47 (3.1%)
NH Black	882 (26.1%)	524 (27.3%)	358 (23.2%)
NH White	2,322 (68.8%)	1,257 (67.5%)	1,065 (70.4%)
NH other races^b^	50 (1.5%)	29 (1.6%)	21 (1.4%)
Unknown	87	58	29
Age at diagnosis			
Mean (SD)	64.5 (12.4)	65.2 (12.4)	63.6 (12.3)
Median (IQR)	65.0 (57.0, 73.0)	66.0 (57.0, 74.0)	64.0 (56.0, 72.0)
Year of diagnosis			
2014	511 (14.8%)	318 (16.6%)	193 (12.5%)
2015	580 (16.8%)	334 (17.4%)	246 (16.0%)
2016	589 (17.0%)	320 (16.7%)	269 (17.5%)
2017	648 (18.7%)	348 (18.1%)	300 (19.5%)
2018	581 (16.8%)	307 (16.0%)	274 (17.8%)
2019	552 (15.9%)	293 (15.3%)	259 (16.8%)
Cancer type			
Breast cancer	398 (11.5%)	305 (15.9%)	93 (6.0%)
Colorectal cancer	501 (14.5%)	267 (13.9%)	234 (15.2%)
NSCLC	1,835 (53.0%)	757 (39.4%)	1,078 (70.0%)
Squamous cell carcinoma	276 (15.0%)	185 (24.4%)	91 (8.4%)
Adenocarcinoma	1,231 (67.1%)	388 (51.3%)	843 (78.2%)
Other NSCLC	328 (17.9%)	184 (24.3%)	144 (13.4%)
Prostate cancer	727 (21.0%)	591 (30.8%)	136 (8.8%)
Cancer recurrence			
Yes	106 (3.9%)	58 (3.9%)	48 (4.0%)
No	2,600 (96.1%)	1,440 (96.1%)	1,160 (96.0%)
Unknown	755	422	333
Gender			
Male	1,945 (56.2%)	1,147 (59.7%)	798 (51.8%)
Female	1,516 (43.8%)	773 (40.3%)	743 (48.2%)
Insurance status at diagnosis			
Private	1,026 (29.6%)	495 (25.8%)	531 (34.5%)
Medicaid	204 (5.9%)	118 (6.1%)	86 (5.6%)
Medicare	1,627 (47.0%)	919 (47.9%)	708 (45.9%)
Other insurance	517 (14.9%)	334 (17.4%)	183 (11.9%)
Uninsured	87 (2.5%)	54 (2.8%)	33 (2.1%)
Rural–urban			
Isolated small rural	2,463 (77.9%)	1,357 (78.1%)	1,106 (77.6%)
Small rural	395 (12.5%)	217 (12.5%)	178 (12.5%)
Large rural/city/town	244 (7.7%)	122 (7.0%)	122 (8.6%)
Urban	61 (1.9%)	42 (2.4%)	19 (1.3%)
Unknown	298	182	116
Proportion of census tract with ≤ high school education, quintiles			
1 (lowest proportion low education)	584 (18.6%)	350 (20.3%)	234 (16.6%)
2	417 (13.3%)	243 (14.1%)	174 (12.4%)
3	540 (17.2%)	302 (17.5%)	238 (16.9%)
4	689 (22.0%)	364 (21.1%)	325 (23.1%)
5 (highest proportion low education)	903 (28.8%)	466 (27.0%)	437 (31.0%)
Unknown	328	195	133
Yost SES index, quintiles			
1 (lowest SES)	750 (23.7%)	376 (21.6%)	374 (26.3%)
2	684 (21.6%)	390 (22.4%)	294 (20.6%)
3	738 (23.3%)	379 (21.8%)	359 (25.2%)
4	609 (19.3%)	368 (21.2%)	241 (16.9%)
5 (highest SES)	381 (12.0%)	225 (12.9%)	156 (11.0%)
Unknown	299	182	117

Abbreviations: IQR, interquartile range; SES, socioeconomic status.

a Percentages exclude missing values.

b NH other races include NH American Indian or Alaska Native, NH Native Hawaiian or Pacific Islander, and NH other races (*n* = 50).

Compared with patients who did not receive genomic testing, patients who did receive genomic testing were younger at diagnosis (median age of 64 vs. 66 years), more likely to be diagnosed in later years (e.g., 16.8% of patients who received genomic testing were diagnosed in 2019 vs. 15.3% of patients who did not receive genomic testing), more likely to be diagnosed with colorectal cancer (15.2% vs. 13.9%) or NSCLC (70.0% vs. 39.4%), particularly adenocarcinoma (78.2% vs. 51.3% of NSCLC diagnoses), and more likely to have private insurance (34.5% vs. 25.8%; Table 1).

### Genomic testing by race and ethnicity and cancer type

The use of genomic testing varied by race and ethnicity within cancer type (Table 2). The proportion of patients tested was generally highest among NSCLC and colorectal cancer—cancer types with more actionable genomic targets during the study period. Testing was particularly high among NH Asian patients with NSCLC (76.1%), colorectal cancer (81.8%), or breast cancer (33.3%) compared with other racial or ethnic groups with those cancers. NH Black patients with colorectal cancer or NSCLC had similar overall genomic testing proportions as NH White patients, although NH Black patients with breast or prostate cancers had lower genomic testing proportions compared with NH White patients (16.8% vs. 26.5% and 12.9% vs. 21.2%, respectively).

**Table 2:** Percentage of patients who used genomic testing by cancer type, race/ethnicity, and type of test

	Any genomic testing	Commercial CGP	In-house NGS^a^	Single-gene testing^b^
	%	%	%	%
Breast cancer				
Hispanic	16.7	16.7	—	—
NH Asian	33.3	33.3	—	—
NH Black	16.8	16.8	—	—
NH White	26.5	26.5	—	—
NH other races	30.0	30.0	—	—
Colorectal cancer				
Hispanic	62.5	37.5	18.8	25.0
NH Asian	81.8	54.6	27.3	18.2
NH Black	46.0	20.6	15.1	14.3
NH White	46.3	29.8	12.1	13.4
NH other races	35.7	21.4	0.0	14.3
NSCLC				
Hispanic	64.3	21.4	42.9	35.7
NH Asian	76.1	41.3	54.4	52.2
NH Black	58.4	26.7	43.5	30.1
NH White	58.6	23.2	42.3	32.3
NH other races	64.7	41.2	52.9	17.7
Prostate cancer				
Hispanic	7.7	7.7	—	—
NH Asian	12.5	12.5	—	—
NH Black	12.9	12.9	—	—
NH White	21.2	21.2	—	—
NH other races	22.2	22.2	—	—

Abbreviations: CGP, comprehensive genomic profiling; NGS, next-generation sequencing; NHW, non-Hispanic White.

a In-house NGS testing was only conducted among colorectal cancer and NSCLC during the study years at our institution.

b Single-gene testing was only conducted among colorectal cancer and NSCLC during the study years at our institution. Tests examined were KRAS, NRAS, and BRAF for colorectal cancer and BRAF, KRAS, EGFR, ALK, RET, and ROS1 for NSCLC.

### Odds of genomic testing by race and ethnicity

In unadjusted analysis stratified by cancer type (only including NH Black and NH White patients due to small sample sizes among other races and ethnicities), NH Black patients with prostate cancer (OR, 0.52, 95% CI, 0.31–0.86) were less likely to receive genomic testing (Table 3), as were NH Black patients diagnosed with breast cancer in the earlier study years of 2014 to 2016 (OR, 0.34, 95% CI, 0.12–0.95; Supplementary Table S1A). This association remained statistically significant for prostate cancer after adjustment for patient- and census-level covariates (fully adjusted OR, 0.55, 95% CI, 0.32–0.95; Table 3). The association for breast cancer remained statistically significant after adjustment for patient-level covariates (OR, 0.34–95% CI, 0.11–0.99) but not after additional adjustment for census-level covariates, although the estimate was imprecise (OR, 0.50, 95% CI, 0.15, 1.64; Supplementary Table S1A). NH Black patients with colorectal cancer or NSCLC were no less likely to receive genomic testing as compared with NH White patients (Table 3). In sensitivity analysis, lower genomic testing among NH Black patients with prostate cancer was observed when limited to patients diagnosed in later years (2017–2019), when limited to patients who survived at least 120 days after diagnosis, and when limited to patients who received any cancer treatment at our institution (Supplementary Table S1B). No statistically significant associations were observed in sensitivity analyses among patients with colorectal cancer (Supplementary Table S1C) or NSCLC (Supplementary Table S1D).

**Table 3:** Regression analysis evaluating the association between any genomic testing and race and ethnicity among NH Black and NH White patients with stage IV cancer diagnosed during 2014 to 2019, stratified by cancer type

	Breast cancer (*n* = 327)				Colorectal cancer (*n* = 390)			
	*N* ^a^	Unadjusted OR (95%CI)	Adjusted for patient-level covariates^b^	Adjusted for patient- and census-level covariates^c^	*N* ^a^	Unadjusted OR (95%CI)	Adjusted for patient-level covariates^b^	Adjusted for patient- and census-level covariates^c^
			OR (95%CI)	OR (95%CI)			OR (95%CI)	OR (95%CI)
NHW	216	1.	1.	1.	277	1.	1.	1.
NHB	111	0.59 (0.33–1.05)	0.65 (0.36–1.19)	0.61 (0.32–1.15)	113	0.92 (0.59–1.42)	1.05 (0.66–1.68)	1.07 (0.67–1.73)

	NSCLC (*n* = 1,580)				Prostate cancer (*n* = 612)			
	*N* ^a^	Unadjusted OR (95%CI)	Adjusted for patient-level covariates^b^	Adjusted for patient- and census-level covariates^c^	*N* ^a^	Unadjusted OR (95%CI)	Adjusted for patient-level covariates^b^	Adjusted for patient- and census-level covariates^c^
			OR (95%CI)	OR (95%CI)			OR (95%CI)	OR (95%CI)
NHW	1,180	1.	1.	1.	427	1.	1.	1.
NHB	400	1.06 (0.84–1.34)	1.08 (0.85–1.38)	1.03 (0.80–1.33)	185	0.52 (0.31–0.86)	0.50 (0.30–0.85)	0.55 (0.32–0.95)

Abbreviations: CI, confidence interval; NHB, non-Hispanic Black; NHW, non-Hispanic White.

a Patients missing any covariate data were excluded from unadjusted and adjusted models.

b Patient-level covariates included age at diagnosis (continuous), year of cancer diagnosis (continuous), and insurance status (uninsured, private, Medicaid, Medicare, other insurance).

c Census tract-level covariates included rural–urban categorization based on RUCA codes (isolated small rural or small rural, large rural/city/town, urban), educational attainment (quintiles of the proportion of the census tract age ≥25 years with a high school education or less), and the Yost socioeconomic status (SES) index—an area-based composite measure of SES (quintiles).

## Discussion

In analysis of more than 3,400 patients diagnosed with stage IV breast cancer, colorectal cancer, NSCLC, or prostate cancer in 2014 to 2019 at a comprehensive cancer center in North Carolina, approximately 45% of patients received any tumor-specific genomic testing. Additionally, we found racial disparities in the receipt of genomic testing for NH Black patients with prostate cancer across the study period and for NH Black patients with breast cancer diagnosed in earlier study years. This study adds novel data from a time period when genomic testing technologies, such as NGS approaches, were emerging as promising tools and were being more widely implemented across the United States—a testing that is a critical first step in receiving precision cancer medicine treatment strategies and accessing certain clinical trials (4).

Evidence of racial and ethnic disparities in genetic testing among patients with cancer from previous US-based studies has been mixed, likely attributable to varying study characteristics, including study population, genetic testing outcome, outcome ascertainment, and study period. For instance, in Flatiron EHR data, no disparities in NGS testing were observed between Black and White patients with breast cancer (3), but Black and Hispanic/Latino men with metastatic prostate cancer were less likely to undergo NGS testing compared with White patients (14). We similarly found lower rates of genomic testing among NH Black patients with prostate cancer. Meanwhile, lower rates of testing have been observed for *EGFR*, *ROS1*, NGS, and any genetic testing among non-White patients with NSCLC (3, 10–13), but other studies have found no racial or ethnic differences for *EGFR*, *ALK*, or NGS testing in this patient population (22, 23), including our own study demonstrating no differences in testing between NH Black and NH White patients with NSCLC. Other studies have found lower rates of genetic testing referral, attendance at genetic counseling appointments, and receipt of genetic testing among NH Black patients with colorectal cancer (24, 25). In our study, we did not find any differences in overall genomic testing between NH Black and NH White patients with colorectal cancer.

Clinical practice guidelines (6), insurance reimbursement policies (26), and approved targeted therapies (27) were all undergoing changes throughout our study period (diagnosis years 2014–2019). For instance, genomic testing was recommended for many metastatic NSCLC and colorectal cancers during our study period, although the number of relevant biomarkers was limited, so single-gene testing was still common. Additionally, the first targeted drugs for hormone receptor (HR)-positive, human epidermal growth factor receptor 2 (*HER2*)-negative breast and metastatic castration-resistant prostate cancers for which NGS testing is relevant were approved in May 2019 (*PIK3CA*, alpelisib; ref. 27) and May 2020 [homologous recombination repair gene mutated, olaparib], respectively, and thus were not available for many patients included in our study. Although genomic testing—particularly NGS approaches—may not have been standard of care for all patients in our analysis given clinical guidelines at the time of their diagnosis, genomic testing to guide the use of targeted therapies is the future of cancer medicine (4, 27). Disparities in the use of genomic testing during a time when these technologies were emerging highlight the need to ensure more equitable access to the most innovative advancements in cancer research in the years to come.

Notably, we did not observe any differences in the overall use of genomic testing between NH Black and NH White patients with colorectal cancer or NSCLC across the study period, and, similarly, no differences were observed for patients with breast cancer diagnosed in later years (2017–2019). We hypothesize that racial disparities may be mitigated as genomic testing recommendations became more standard over the study period for certain cancers and thus less subject to barriers at the patient (e.g., insurance coverage) or provider levels (e.g., implicit bias). For instance, it may be the case that NH White patients with breast cancer were more often seeking genomic testing before it was a standard of care as a means to enroll in a clinical trial, so patient awareness and self-advocacy—in addition to barriers to care—may have contributed to racial differences in testing in earlier years.

Several limitations of this study should be considered. Our primary predictor of interest was race and ethnicity, but our sample sizes for patients who were not NH Black or NH White were too small to conduct more comprehensive analysis for those groups. However, our study was conducted in North Carolina—a state with nearly one-quarter of the population identifying as Black (28) and where Black males and females have the highest cancer mortality rates of any racial group (CDC WONDER, RRID:SCR_025830). We also lacked data on more detailed patient-, disease-, and clinic-level factors that may have influenced a provider’s recommendation for genomic testing (e.g., comorbidities that contraindicated therapy, disease prognosis, HR status, tissue availability, and laboratory turnaround times). Additional quantitative and qualitative research is needed to disentangle the multilevel factors that may be driving differences in testing by race and ethnicity. Additionally, we were not able to examine how the use of genomic testing translated into the use of targeted therapies. Lastly, because our data were from a single comprehensive cancer center, our findings may not be generalizable to other institutions.

Our study documents the presence of disparities for NH Black patients with metastatic prostate cancer within a large comprehensive cancer center in North Carolina—an institute which serves a catchment area with a high proportion of Black residents across North Carolina, South Carolina, and Virginia. Data demonstrating the lesser use of tumor-specific genomic testing among NH Black cancer patients—from our study and others (3, 9–13)—should serve as a call to action for clinicians, research scientists, and policymakers to better understand the multitude of factors that likely contribute to such disparities and target efforts to mitigate them. Our study’s documentation of such disparities highlights the importance of monitoring the trends in precision oncology advancements to ensure we are proactive in addressing emerging disparities. Future work to better understand and address barriers to genomic testing at the patient, provider, clinic, and system levels is a crucial next step forward, including patient and provider education, more effective patient–provider communication, increased clinic-level access to testing resources, standardization of the ordering procedure, more efficient laboratory turnaround times, and improved insurance coverage for testing.

## Supplementary Material

Supplementary Table 1 Supplementary Tables S1a-S1d show estimates for the association between any genomic testing and race and ethnicity among non-Hispanic Black and non-Hispanic White patients by cancer type
