# Supplementary material for: Association of Race and Ethnicity with Genomic Testing at a Comprehensive Cancer Center in North Carolina
Source: Cancer Res Commun. 2024 Nov 18;4(11):2968–75. doi: 10.1158/2767-9764.CRC-24-0134 (PMC11570879; doi:10.1158/2767-9764.CRC-24-0134)
Supplement: Supplementary Table 1 — Supplementary Tables S1a-S1d show estimates for the association between any genomic testing and race and ethnicity among non-Hispanic Black and non-Hispanic White patients by cancer type [file crc-24-0134_supplementary_table_1_suppst1.pdf]

Table S1a. Sensitivity analysis evaluating the association between any genomic testing and race and ethnicity among non-Hispanic Black and non-Hispanic White patients with stage IV breast cancer, stratified by clinical characteristics

	Diagnosed 2014-2016 (n=158)				Diagnosed 2017-2019 (n=169)			
	N[a]	Unadjusted OR (95% CI)	Adjusted for patient-level covariates[b] OR (95% CI)	Adjusted for patient- and census-level covariates[c] OR (95% CI)	N[a]	Unadjusted OR (95% CI)	Adjusted for patient-level covariates[b] OR (95% CI)	Adjusted for patient- and census-level covariates[c] OR (95% CI)
NHW	110	1.	1.	1.	106	1.	1.	1.
NHB	48	0.34 (0.12, 0.95)	0.34 (0.11, 0.99)	0.50 (0.15, 1.64)	63	0.80 (0.38, 1.66)	0.94 (0.44, 2.00)	0.74 (0.34, 1.64)
	Survived ≥120 days after diagnosis (n=292)				Received any cancer treatment (n=324)			
	N[a]	Unadjusted OR (95% CI)	Adjusted for patient-level covariates[b] OR (95% CI)	Adjusted for patient- and census-level covariates[c] OR (95% CI)	N[a]	Unadjusted OR (95% CI)	Adjusted for patient-level covariates[b] OR (95% CI)	Adjusted for patient- and census-level covariates[c] OR (95% CI)
NHW	194	1.	1.	1.	214	1.	1.	1.
NHB	98	0.57 (0.31,1.04)	0.57 (0.30, 1.10)	1.03 (0.80, 1.33)	110	0.59 (0.33, 1.05)	0.65 (0.35, 1.18)	0.62 (0.33, 1.16)

Abbreviations: CI, confidence interval; NHB, non-Hispanic Black; NHW, non-Hispanic White; OR, odds ratio

[a]Patients missing any covariate data were excluded from unadjusted and adjusted models.

[b]Patient-level covariates included: age at diagnosis (continuous), year of cancer diagnosis (continuous), and insurance status (uninsured, private, Medicaid, Medicare, other insurance).

[c]Census tract-level covariates included: rural-urban categorization based on rural-urban commuting area (RUCA) codes (isolated small rural or small rural, large rural/city/town, urban), educational attainment (quintiles of the proportion of the census tract age ≥25 years with a high school education or less), and the Yost socioeconomic status (SES) index—an area-based composite measure of SES (quintiles).

Table S1b. Sensitivity analysis evaluating the association between any genomic testing and race and ethnicity among non-Hispanic Black and non-Hispanic White patients with stage IV prostate cancer, stratified by clinical characteristics

	Diagnosed 2014-2016 (n=278)				Diagnosed 2017-2019 (n=334)			
	N[a]	Unadjusted OR (95% CI)	Adjusted for patient-level covariates[b] OR (95% CI)	Adjusted for patient- and census-level covariates[c] OR (95% CI)	N[a]	Unadjusted OR (95% CI)	Adjusted for patient-level covariates[b] OR (95% CI)	Adjusted for patient- and census-level covariates[c] OR (95% CI)
NHW	194	1.	1.	1.	233	1.	1.	1.
NHB	84	0.61 (0.28, 1.34)	0.67 (0.29, 1.55)	0.73 (0.31, 1.72)	101	0.47 (0.24, 0.90)	0.44 (0.22, 0.87)	0.50 (0.24, 1.03)

	Survived ≥120 days after diagnosis (n=586)				Received any cancer treatment (n=603)			
	N[a]	Unadjusted OR (95% CI)	Adjusted for patient-level covariates[b] OR (95% CI)	Adjusted for patient- and census-level covariates[c] OR (95% CI)	N[a]	Unadjusted OR (95% CI)	Adjusted for patient-level covariates[b] OR (95% CI)	Adjusted for patient- and census-level covariates[c] OR (95% CI)
NHW	406	1.	1.	1.	423	1.	1.	1.
NHB	180	0.51 (0.31, 0.85)	0.50 (0.30, 0.85)	0.55 (0.32, 0.95)	180	0.53 (0.32, 0.88)	0.52 (0.30, 0.87)	0.56 (0.32, 0.96)

Abbreviations: CI, confidence interval; NHB, non-Hispanic Black; NHW, non-Hispanic White; OR, odds ratio
[a]Patients missing any covariate data were excluded from unadjusted and adjusted models.
[b]Patient-level covariates included: age at diagnosis (continuous), year of cancer diagnosis (continuous), and insurance status (uninsured, private, Medicaid, Medicare, other insurance).
[c]Census tract-level covariates included: rural-urban categorization based on rural-urban commuting area (RUCA) codes (isolated small rural or small rural, large rural/city/town, urban), educational attainment (quintiles of the proportion of the census tract age ≥25 years with a high school education or less), and the Yost socioeconomic status (SES) index—an area-based composite measure of SES (quintiles).

Table S1c. Sensitivity analysis evaluating the association between any genomic testing and race and ethnicity among non-Hispanic Black and non-Hispanic White patients with stage IV colorectal cancer, stratified by clinical characteristics

	Diagnosed 2014-2016 (n=204)				Diagnosed 2017-2019 (n=186)			
	N[a]	Unadjusted OR (95% CI)	Adjusted for patient-level covariates[b] OR (95% CI)	Adjusted for patient- and census-level covariates[c] OR (95% CI)	N[a]	Unadjusted OR (95% CI)	Adjusted for patient-level covariates[b] OR (95% CI)	Adjusted for patient- and census-level covariates[c] OR (95% CI)
NHW	146	1.	1.	1.	131	1.	1.	1.
NHB	58	1.00 (0.54, 1.84)	1.39 (0.70, 2.77)	1.51 (0.73, 3.11)	55	0.83 (0.44, 1.56)	0.87 (0.45, 1.69)	0.90 (0.46,1.79)

	Survived ≥120 days after diagnosis (n=322)				Received any cancer treatment (n=381)			
	N[a]	Unadjusted OR (95% CI)	Adjusted for patient-level covariates[b] OR (95% CI)	Adjusted for patient- and census-level covariates[c] OR (95% CI)	N[a]	Unadjusted OR (95% CI)	Adjusted for patient-level covariates[b] OR (95% CI)	Adjusted for patient- and census-level covariates[c] OR (95% CI)
NHW	233	1.	1.	1.	274	1.	1.	1.
NHB	89	1.19 (0.73, 1.95)	1.31 (0.78, 2.20)	1.39 (0.82, 2.37)	107	0.99 (0.64, 1.56)	1.14 (0.71, 1.84)	1.16 (0.71, 1.88)

Abbreviations: CI, confidence interval; NHB, non-Hispanic Black; NHW, non-Hispanic White; OR, odds ratio

[a]Patients missing any covariate data were excluded from unadjusted and adjusted models.

[b]Patient-level covariates included: age at diagnosis (continuous), year of cancer diagnosis (continuous), and insurance status (uninsured, private, Medicaid, Medicare, other insurance).

[c]Census tract-level covariates included: rural-urban categorization based on rural-urban commuting area (RUCA) codes (isolated small rural or small rural, large rural/city/town, urban), educational attainment (quintiles of the proportion of the census tract age ≥25 years with a high school education or less), and the Yost socioeconomic status (SES) index—an area-based composite measure of SES (quintiles).

Table S1d. Sensitivity analysis evaluating the association between any genomic testing and race and ethnicity among non-Hispanic Black and non-Hispanic White patients with stage IV non-small cell lung cancer, stratified by clinical characteristics

	Diagnosed 2014-2016 (n=784)				Diagnosed 2017-2019 (n=796)			
	N[a]	Unadjusted OR (95% CI)	Adjusted for patient-level covariates[b] OR (95% CI)	Adjusted for patient- and census-level covariates[c] OR (95% CI)	N[a]	Unadjusted OR (95% CI)	Adjusted for patient-level covariates[b] OR (95% CI)	Adjusted for patient- and census-level covariates[c] OR (95% CI)
NHW	598	1.	1.	1.	582	1.	1.	1.
NHB	196	0.90 (0.65, 1.25)	0.86 (0.61, 1.22)	0.88 (0.61, 1.28)	214	1.22 (0.88, 1.69)	1.32 (0.94, 1.86)	1.08 (0.75, 1.56)

	Survived ≥120 days after diagnosis (n=1139)				Received any cancer treatment (n=1518)			
	N[a]	Unadjusted OR (95% CI)	Adjusted for patient-level covariates[b] OR (95% CI)	Adjusted for patient- and census-level covariates[c] OR (95% CI)	N[a]	Unadjusted OR (95% CI)	Adjusted for patient-level covariates[b] OR (95% CI)	Adjusted for patient- and census-level covariates[c] OR (95% CI)
NHW	855	1.	1.	1.	1137	1.	1.	1.
NHB	284	1.09 (0.82, 1.45)	1.14 (0.85, 1.54)	1.10 (0.81, 1.51)	381	1.08 (0.86, 1.38)	1.11 (0.87, 1.42)	1.07 (0.82, 1.38)

Abbreviations: CI, confidence interval; NHB, non-Hispanic Black; NHW, non-Hispanic White; OR, odds ratio

[a]Patients missing any covariate data were excluded from unadjusted and adjusted models.

[b]Patient-level covariates included: age at diagnosis (continuous), year of cancer diagnosis (continuous), and insurance status (uninsured, private, Medicaid, Medicare, other insurance).

[c]Census tract-level covariates included: rural-urban categorization based on rural-urban commuting area (RUCA) codes (isolated small rural or small rural, large rural/city/town, urban), educational attainment (quintiles of the proportion of the census tract age ≥25 years with a high school education or less), and the Yost socioeconomic status (SES) index—an area-based composite measure of SES (quintiles).
